# Pneumopericardium, Epidural Pneumatosis, and Muscular Emphysema: Rare Complications of Spontaneous Pneumomediastinum Due to Refractory Hyperemesis Gravidarum

**DOI:** 10.7759/cureus.23800

**Published:** 2022-04-04

**Authors:** Charles R Russell, Garnett Benjamin, Joshua K Salabei, Peters Okonoboh, Liang Sun

**Affiliations:** 1 Internal Medicine, University of Central Florida College of Medicine/HCA Florida North Florida Hospital, Gainesville, USA; 2 Medicine, Trinity School of Medicine, Roswell, USA; 3 Internal Medicine - Critical Care, University of Central Florida College of Medicine/HCA Florida North Florida Hospital, Gainesville, USA; 4 Internal Medicine, HCA Florida North Florida Hospital, Gainesville, USA

**Keywords:** spontaneous pneumothorax, cannabinoids, subcutaneous emphysema, spontaneous pneumomediastinum (spm), hyperemesis gravidarum

## Abstract

Various complications of hyperemesis gravidarum and pneumomediastinum have been documented in the literature. Commonly, these cases resolve spontaneously or with the use of antiemetics and supportive care. In rare instances, these symptoms persist into the second trimester and are associated with an increased risk of complications. Herein, we present a case of a healthy 20-year-old female with a history of marijuana use who presented with intractable nausea and vomiting and was found to have multiple rare complications of spontaneous pneumomediastinum.

## Introduction

Pneumomediastinum has been documented as a possible complication in conditions associated with high intrathoracic pressure such as tension pneumothorax, intractable coughing, or a rupture to the esophagus or respiratory system [1]. The condition is more commonly seen in young male patients and pregnant patients during the second stage of labor [1]. Less commonly it can be associated with hyperemesis gravidarum (HG) [2,3]. Spontaneous pneumomediastinum is usually a self-limiting condition commonly associated with subcutaneous emphysema, but can have dangerous complications such as pneumopericardium, pneumothorax, and rarely epidural pneumatosis in a protracted case [1-6]. It is well understood that pneumomediastinum occurs much more frequently in males than females, accounting for over 75% of cases [1]. Here we discuss a particularly and exceedingly rare case of refractory HG with three simultaneously occurring rare complications of spontaneous pneumomediastinum in an otherwise healthy 20-year-old female.

## Case presentation

A 20-year-old gravida (G) 1, para (P) 0 female at 12 weeks of gestation presented to the emergency department with a six-week history of intractable nausea, vomiting, and a 20 lb weight loss. On admission, she complained of chest pain and a swollen face. Of note, her past medical history was positive for recent diagnosis of hyperemesis gravidarum six weeks prior. She visited the emergency department five times within six weeks for the same symptoms, and was treated with an assortment of anti-nausea medications including ondansetron, promethazine, metoclopramide, and diphenhydramine without long-lasting relief. Her past medical history was positive for marijuana for which the patient’s labs were positive during current admission. The patient denied using marijuana after finding out she was pregnant.

**Figure 1:**
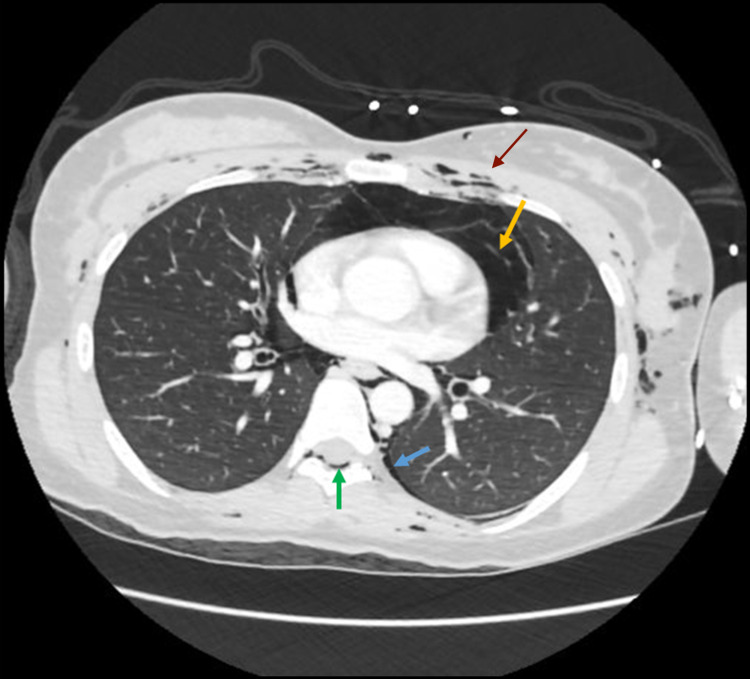
Computed tomography of the chest showing pneumothorax (blue arrow), pneumopericardium (yellow arrow), epidural pneumorrhachis (green arrow), and muscular emphysema (red arrow)

**Figure 2:**
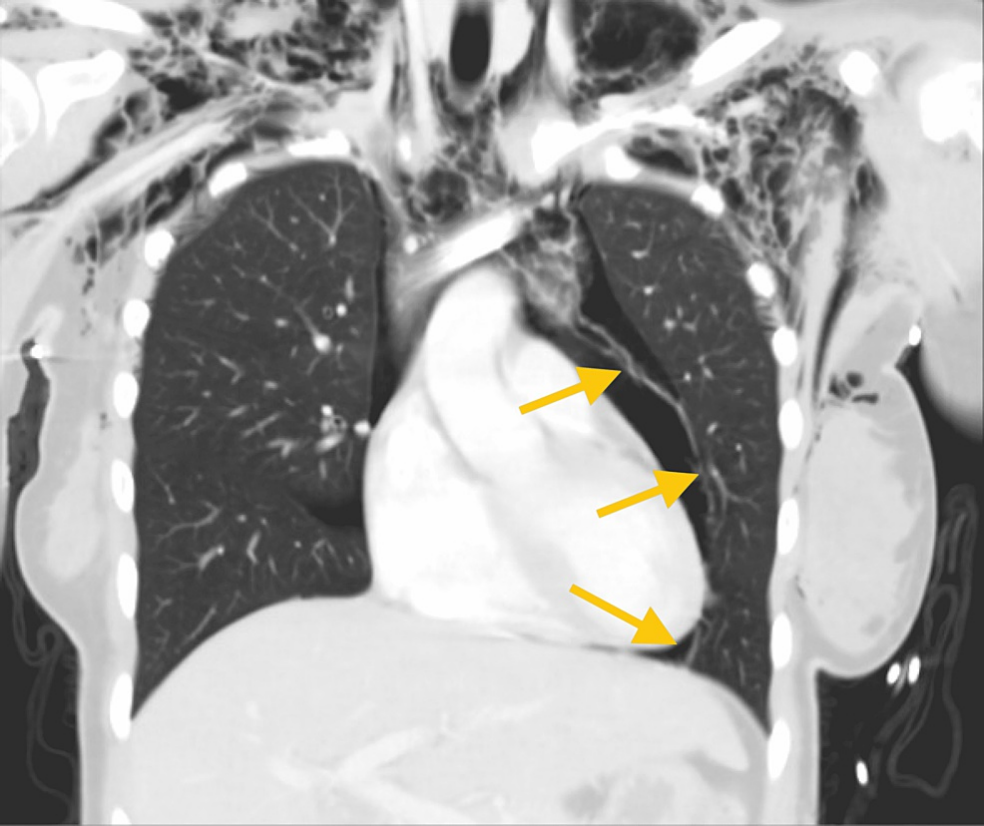
Coronal computed tomography of the chest further demonstrating pneumopericardium (yellow arrows)

**Figure 3:**
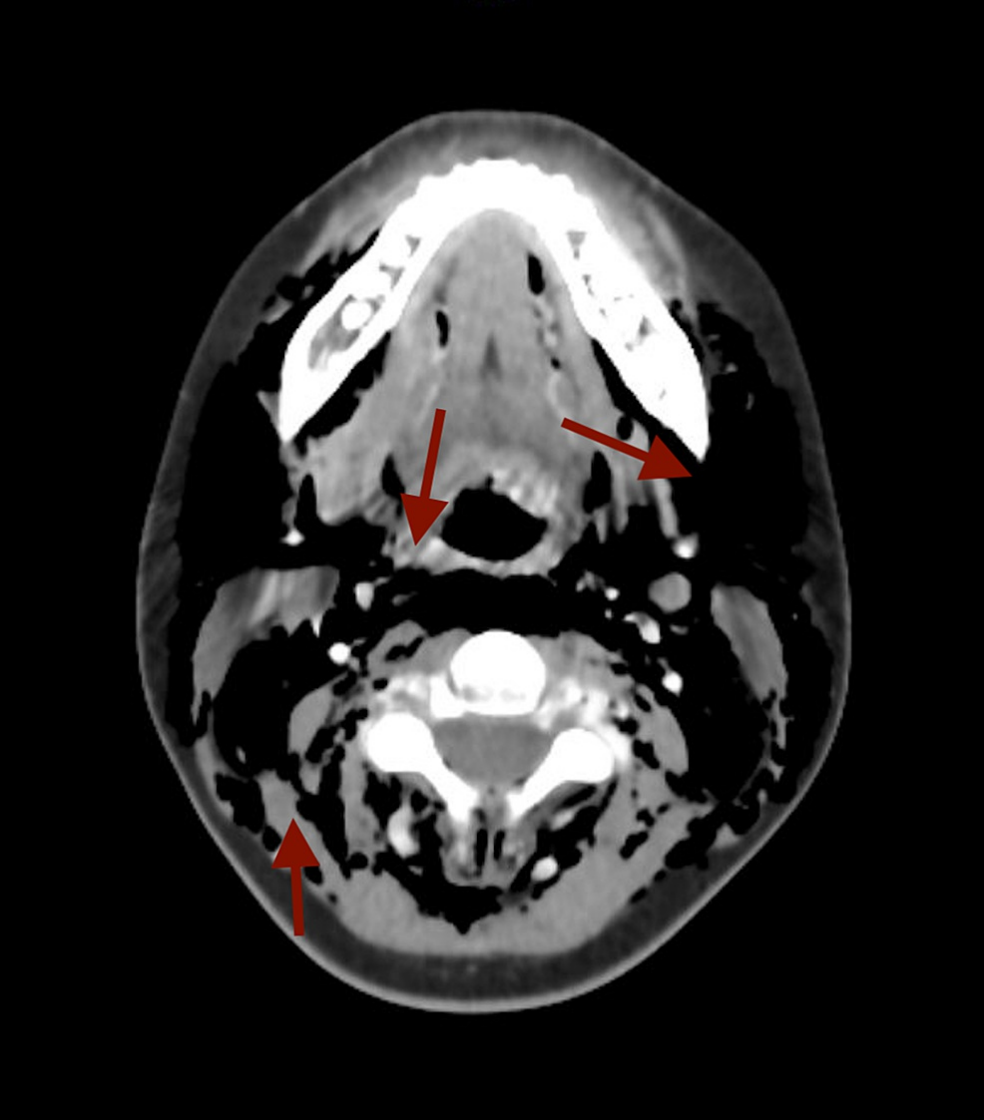
Computerized tomography of the neck showing pneumomediastinum with subcutaneous gas throughout the neck soft tissue (red arrows)

On examination, her vital signs were as follows: blood pressure 165/97 mmHg, pulse rate 114 beats/min, respiratory rate 25 breaths/min, temperature 36.6, with O_2_ saturation 98% on room air. Her physical examination revealed facial swelling and extensive palpable subcutaneous emphysema beginning in the face with periorbital/periocular involvement and extending down to the neck, chest, and abdomen. Pertinent laboratory values were as follows: sodium 132 mmol/L, potassium 2.4 mmol/L, chloride 91 mmol/L, elevated transaminase (aspartate aminotransferase 133 IU/L, alanine aminotransferase 332 IU/L), troponin I <0.015 ng/mL, and elevated globulin 5.0 g/dL. A quantitative beta-human chorionic gonadotropin (hCG) test showed an hCG level of 175,838 mIU/mL increased from 166,774 mIU/mL one week prior. Urinalysis revealed 30-40 white blood cells/high power field, 1+ leukocyte esterase, and positive nitrite. An electrocardiogram (EKG) was ordered to evaluate chest pain and displayed nonspecific T wave abnormalities and sinus tachycardia. Chest X-ray revealed air in the mediastinum and chest wall emphysema indicative of pneumomediastinum. Computerized tomography (CT) of the chest further demonstrated an enormous amount of pneumomediastinum with soft tissue and musculature emphysema anteriorly, laterally, and posteriorly and revealed free air around the pericardium representing pneumopericardium (Figures 1, 2). Furthermore, there were trace amounts of air visualized on the CT image likely representing small bilateral pneumothoraces with large-scale epidural pneumatosis involving the cervical and thoracic regions of the spinal cord (Figure 1). CT of the neck revealed massive pneumomediastinum with subcutaneous gas throughout the neck soft tissue without a focal source (Figure 3). There was extensive soft tissue gas extending into the face on the left more than right involving the left preseptal space.

Supportive care was initiated with intravenous administration of intravenous crystalloids and aggressive repletion of electrolyte deficiencies with continued close monitoring. She was found positive for systemic inflammatory response syndrome (SIRS) criteria due to leukocytosis, tachycardia, and tachypnea with a lactate level of 3.6 mmol/L. Due to the presentation of severe sepsis with suspected intra-abdominal/urinary source, the patient was started on empiric piperacillin/tazobactam 3.175 mg every eight hours. Symptomatic relief was attempted via multimodal approach of diphenhydramine 50 mg, ondansetron 4 mg, and metoclopramide 10 mg. An ear, nose, throat (ENT) specialist was consulted to evaluate neck emphysema and possible pharyngeal rupture. Gynecology was consulted and medical management with a close follow-up was suggested. Surgery was consulted and an upper gastrointestinal (GI) study with gastrograffin was ordered to evaluate for esophageal rupture that revealed no focal source. In light of the unrevealing gastrograffin study, they recommended continued supportive care with symptomatic relief and antibiotic prophylaxis to prevent mediastinitis. On day 4 of admission, facial swelling was improved, but the patient’s intractable nausea/vomiting had not abated with the use of multiple antiemetics. Repeat chest X-ray revealed unchanged pneumomediastinum with persistent subcutaneous emphysema and pneumopericardium. Gynecology recommended the termination of the pregnancy due to extenuating circumstances and failure to control nausea/vomiting with medical therapy; ultimately, the patient agreed to having a termination via suction dilatation and curettage (D&C). On day 5 post-D&C, patient’s symptoms had quickly abated and her electrolyte imbalance and transaminase levels began to improve. The patient was subsequently discharged on day 6 with a seven-day course of amoxicillin/clavulanate potassium 875 mg-125 mg and probiotics for mediastinitis prophylaxis.

## Discussion

Subcutaneous emphysema is a typical sign seen in traumatic injuries caused by air escaping from a ruptured respiratory organ or diffusion from an area of extremely high intrathoracic pressure [1]. Rarely in cases of HG is there any evidence of traumatic injury leading to air dissecting into the mediastinum [1]. It has been suggested that the more likely cause of pneumomediastinum is either increased pressure on the alveolar sacs leading to rupture or air diffusing into the mediastinum due to an increased pressure gradient within the thorax [1]. Typically, patients who have HG present within the first trimester and are managed conservatively with antiemetics [3]. In our patient, the etiology for HG was not fully understood but increased beta-hcg levels have been implicated as a possible cause [4]. In our case, the patient’s beta-hCG levels were triple the expected levels at six weeks of gestation. Additionally, the chronic use of cannabinoids has been associated with the development and prolonged course of hyperemesis syndromes [1,7]. Our patient’s positive urine drug test at least four weeks after her stated stop date is an indication of either chronic or current marijuana use.

To our knowledge, the development of pneumopericardium has not been documented previously in a case of hyperemesis gravidarum, and it predisposes patients to dangerous complications such as cardiac tamponade and mediastinitis [5]. Cases with this presentation can quickly deteriorate and benefit from treatment of the precipitating condition and prophylaxis to prevent mediastinitis.

Epidural pneumatosis is typically a benign condition most commonly associated with the administration of an epidural anesthetic [5,6]. Its course is usually self-limited without a need for additional treatment [5,6]. However, in rare instances, epidural pneumatosis leads to the compression of the spinal cord resulting in focal deficits [6]. While these symptoms have been reported as transient events, it is imperative for the physician to be aware of this potentially fatal complication.

This case is meant to show the dangers of refractory HG. Patients treated conservatively with antiemetics are typically successfully managed and discharged with no sequela. In patients who are not well managed medically, there is a significant increase in the risk of morbidity and mortality [2]. In our case, this patient was seen six times in six weeks for the same symptoms without resolution. For risk reduction and symptom resolution, it is imperative to discuss the risks and benefits of termination with these patients.

## Conclusions

In conclusion, we have presented a rare case of refractory HG complicated by pneumopericardium and epidural pneumorrhachis. Although these cases are rare, clinicians must be vigilant in monitoring patients who experience extended periods of nausea and vomiting, which do not remit after the administration of antiemetics or other forms of conservative therapy. These patients can be at risk of fatal complications such as pneumopericardium. Therefore, clinicians should maintain a high level of suspicion when managing patients with refractory HG.
